# Mitochondrial pyruvate carrier: a metabolic-epigenetic checkpoint orchestrating T cell differentiation

**DOI:** 10.1038/s41392-022-01101-z

**Published:** 2022-08-12

**Authors:** Yu Chen, Jingchun Wang, Yongsheng Li

**Affiliations:** 1 Department of Medical Oncology, Chongqing University Cancer Hospital, 400030 Chongqing, China; 2 Department of Gastroenterology, Xinqiao Hospital, Army Medical University, 400037 Chongqing, China

**Keywords:** Tumour immunology, Cancer metabolism

In a recent study published in *Cell Metabolism*, Wenes et al.^1^ identified that the inhibition of mitochondrial pyruvate carrier (MPC) led to CD8^+^ memory T cell (T_mem_) differentiation and facilitated superior and long-lasting anti-tumor activity of chimeric antigen receptor (CAR) T cells, which suggests a potential target for enhancing anti-tumor immunity.

**Fig. 1:**
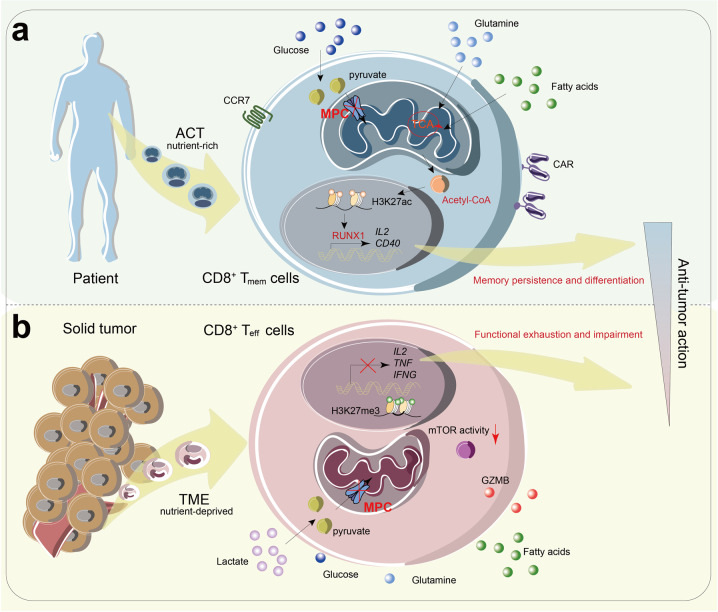
MPC regulates memory CD8^+^ T cells differentiation and anti-tumor effect in adoptive cell therapy and tumor microenvironment. **a** In adoptive cell therapy, MPC deletion blocks pyruvate metabolism in CAR T cells, which facilitates the oxidation of fatty acids and glutamine in TCA. The increased acetyl-CoA enhances histone acetylation and chromatin accessibility of transcription factor RUNX1, which up-regulates pro-memory genes to promote the development of CD8+ memory T cells (Tmem), resulting in increased persistence and anti-tumor effect of CAR T cells. **b** In nutrient-limited TME, CD8^+^ T cells rely on lactate oxidation. MPC deficiency blunts pyruvate utilization and causes mTOR inactivation and H3K27 methylation, and impairs the anti-tumor function of CD8^+^ effector T cells (T_eff_)

Metabolic reprogramming drives CD8^+^ T cell development and function. The glucose-derived pyruvate utilization is essential for CD8^+^ T cell activation and differentiation into effector cells,^2^ whereas the underlying molecular basis of mitochondrial pyruvate import on T cell differentiation remains largely unknown. MPC is the transporter mediating cytosolic pyruvate entering the mitochondrial matrix, thereby promoting pyruvate oxidation. Pioneering studies have reported that MPC-dependent mitochondrial pyruvate uptake was crucial for maintaining homeostatic T cell development, and deletion of MPC in early thymic development results in reduced numbers and abnormal activation of peripheral αβ T cells.^3^ In this study, Wenes et al. showed that MPC-deficient T cells displayed memory-skewing features, i.e., formed more memory precursor effector cells (MPECs) and central memory T cells during their activation and expansion (Fig. 1).^1^ MPC deficiency blunted the anti-tumor potential of tumor-infiltrating lymphocytes (TILs) by blocking lactate oxidation in mitochondria. Thus blocking mitochondrial pyruvate uptake promoted memory differentiation of CD8^+^ T cells.

Acetyl-coenzyme-A (acetyl-CoA) is an intermediate metabolic hub for glucose, lipids and amino acids. Given that inhibiting the mitochondrial uptake of glucose-derived pyruvate increases glutamine oxidation and fatty acid oxidation (FAO) in several cell lines, Wenes et al. used uniformly ^13^C-labeled glucose, glutamine, or palmitate to determine the changes of metabolic pathways after inhibiting MPC. Consistently, MPC inhibitor (MPCi) suppressed glucose incorporation in tricarboxylic acid cycle (TCA) while increased the oxidation of fatty acid and glutamine, resulting in a significant increase in the overall levels of acetyl-CoA in CD8^+^ T cells.^1^

It is well-known that acetyl-CoA is the substrate for histone acetylation, a critical epigenetic modification. Notably, the acetylation of lysine residue 27 on histone H3 (H3K27ac), which was reported closely correlated with CD8^+^ T_mem_ differentiation,^4^ was increased by MPCi-induced acetyl-CoA accumulation. This work revealed that MPCi activated IL-2 and CD40 signaling, the crucial pathways for T_mem_ differentiation, following the metabolic-epigenetic axis was dependent on RUNX1 coordination. Additionally, in a nutrient-deprived tumor microenvironment (TME), abundant lactate can be metabolized to produce pyruvate in TILs and subsequently be transported into mitochondria to be completely oxidized. The authors revealed that lactate oxidation was essential for preventing the loss of mTOR signaling and the chromatin-condensing trimethylation of H3K27 (H3K27m3) resulted in maintaining the function of CD8^+^ effector T cells (T_eff_)^4^ (Fig. 1). Consistently, MPC knockout (KO) T cells displayed lower expression of IL-2, IFN-γ, and TNF in the present of low glucose, glutamine and high lactate, compared with WT (wild-type) T cells in vitro. Interestingly, the transfer of MPC KO CD8^+^ T cells into tumor-bearing mice barely reduced tumor growth as compared with untreated mice, whereas WT CD8^+^ T cells did, which was related with the downregulated anti-tumor function of CD8^+^ T cells mediated by the impede of lactate oxidation. Therefore, at least in the tumor-bearing models tested in this study,^1^ MPC-mediated mitochondrial pyruvate import is required for sustaining the anti-tumor effector function of TILs.

The adoptive cell therapy (ACT) is one of the major forms of cancer immunotherapy, but some patients have gained slight or no benefit from it due to low expansion, persistence and continued activity of the adoptively transferred cells. The CAR T cells that possess and maintain less differentiated phenotypes, such as CD8^+^ T_mem_ cells, are critical for anti-tumor efficacy and patient outcomes. The metabolic reprogramming and epigenetic modification orchestrates persistence and survival of CD8^+^ T_mem_ cells. IL-7 and IL-15 promote T cells survival and memory development through facilitating glucose uptake and FAO. CAR T cells manufactured by these two interleukins are currently ongoing Phase IV clinical trials for B cell lymphoma treatment.^5^ In this article, Wenes et al. discovered that transient usage of MPCi during CAR T production amplified its anti-tumor efficacy and persistence. The acetylation on histone H3K27 was significantly induced after MPCi treatment. Human CAR T cells with MPCi also improved the survival in xenograft leukemia mouse model.^1^ Therefore, blockade of MPC in nutrient-rich environment may be a promising approach to generate more efficient CAR T cells.

Beyond this research, several unresolved questions await further investigations. Firstly, the concrete mechanism between metabolic reprogramming and RUNX1 remains vague. Secondly, the metabolic pattern is critical for CD8^+^ T_mem_ cells differentiation. It should be confirmed whether the inhibition of MPC influences the mitochondrial function and pyruvate metabolic pathway. Thirdly, in addition to be derived from glutamine oxidation and FAO, acetyl-CoA can be produced from other alternative pathways which may also partially regulate T cell differentiation. Of note, MPC transient inhibitor UK5099 is also an inhibitor of mono-carboxylate transporter (MCT), its side effect was not referred in this research. It is a potential research direction to find a more specific and safer inhibitor of MPC.

To sum up, this study illustrated that MPC-mediated mitochondrial pyruvate import regulates the metabolic flexibility of CD8^+^ T_mem_ cells and suggested MPC as a new pharmacological molecular target to facilitate superior and long-lasting anti-tumor efficacy of CAR T cells. Mechanistically, the acetyl-CoA production by the glutamine oxidation and FAO were promoted by MPC inhibition, which resulted in enhanced histone acetylation and chromatin accessibility of transcription factor RUNX1 to pro-memory genes. However, MPC deficiency of T cells in the TME with low glutamine and fatty acids decreased mTOR signaling and promoted histone trimethylation, which impaired the anti-tumor potency of T cells. These findings demonstrate that the mitochondria pyruvate uptake controls T cell differentiation and activation in tumor immunity. In translational level, this study indicates that MPC activation in tumor-infiltrated T cells may be a novel approach to promote lactate utilization and the anti-tumor efficacy. Targeting MPC enhances the anti-tumor action, memory phenotype and persistence of CAR T cells, which suggests a potential strategy to improve CAR T cell immunotherapy in future clinical trials.
